# Basic-patch mutations in bacteriophage T4 Rad50 uncouple ATPase activation from processive nuclease activity

**DOI:** 10.1042/BSR20260367

**Published:** 2026-07-23

**Authors:** Tibebe A. Teklemariam, Ryan M. Finnerty, Jennifer B. Coats, Scott W. Nelson

**Affiliations:** 1 Roy J. Carver Department of Biochemistry, Biophysics, and Molecular Biology, Iowa State University, Ames, IA 50011, U.S.A.; 2 Division of Oncology, Washington University School of Medicine, Washington University., 660 S. Euclid Ave., St. Louis, MO 63110, U.S.A.; 3 University of Colorado Anschutz Medical Campus, Aurora, CO 80045, U.S.A.; 4 Department of Chemistry, University of Michigan, 930 N. University, Ann Arbor, MI 48109, USA.

**Keywords:** Allostery, ATPase activity, Bacteriophage T4, Mre11, Nuclease activity, Rad50

## Abstract

The Mre11/Rad50 (MR) complex uses adenosine triphosphate (ATP) binding and hydrolysis to coordinate the recognition and processing of DNA double-strand breaks. Although Mre11 and DNA stimulate the relatively slow ATPase activity of Rad50, the mechanism by which this occurs remains incompletely understood. In the present study, we investigated a basic patch on bacteriophage T4 Rad50, consisting of Arg154, Arg155, and Lys156, that was predicted to contribute to DNA binding. Mutation of these residues caused only modest changes in DNA affinity, indicating that this region is unlikely to function primarily as a direct DNA-contact surface. In contrast, the effects on ATP hydrolysis were pronounced. R154A and the TripleA mutant displayed strong ATPase activation in the presence of Mre11 alone, approaching the activity of the wild-type MR complex bound to DNA. DNA titrations further showed that these mutants were relatively insensitive to increasing double-stranded DNA concentrations, consistent with a shift in the conformational equilibrium toward an ATPase-active-like state. However, ATP-dependent stimulation of repetitive nucleotide excision was reduced for all mutants, with the strongest defect observed for TripleA, indicating that enhanced ATP hydrolysis alone is not sufficient to support processive nuclease activity. A mutation at Asp479 had a related but distinct effect, supporting long-range coupling within the T4 MR complex. Overall, the results support a model in which a basic patch near the base of the Rad50 coiled-coils contributes to an allosteric pathway linking Mre11 and DNA engagement with productive ATP hydrolysis and its coupling to nuclease output.

## Introduction

DNA double-strand breaks (DSBs) are among the most severe lesions that can occur in a cell. Ionizing radiation and a variety of anticancer drugs are capable of inducing DSBs either directly or indirectly. Furthermore, DSBs are regularly generated as a result of defects in DNA replication, for instance, when replicative forks collapse as they attempt to replicate across nicks, lesions, and interstrand crosslinks [1–3]. At the cellular level, the effects of DSBs can manifest in the form of altered proliferation and differentiation, as well as apoptosis. These occurrences are integral components of the DNA damage response and the process of repairing DSBs [4].

In most organisms, DSBs are repaired through homologous recombination (HR), which uses a homologous DNA template for the accurate repair of the DSBs. HR is especially important for the repair of single-ended DSBs that result from collapsed replication forks [5–7]. A 5′ recessed DSB with a 3′ overhang is the hallmark intermediate of HR, and the resection of a DSB is thought to be one of the factors that commit the DSB to HR [5,6,8,9]. In eukaryotes, archaea, and some bacteriophages, the Mre11–Rad50 complex (MR), along with accessory factors, participates in DSB recognition and initial nucleolytic processing [10]. The endonuclease activity of Mre11 introduces a single-strand cut upstream of DSB and proceeds toward the 5′ end using its 3′ to 5′ exonuclease activity [11]. This action removes any protein that may be bound to the DSB and generates a 3′ single-stranded DNA (ssDNA) overhang that in eukaryotes acts as an entry point for ExoI and Dna2, which proceed to carry out extensive DNA resection in the 5′ to 3′ direction [12].

Mre11 is a Mn^2+^-dependent 3′–5′ exonuclease, and it also possesses 5′ ssDNA endonuclease and hairpin nuclease activities. The structure of Mre11 is organized into an N-terminal nuclease domain, a capping domain that confers substrate specificity and access, and a C-terminal Rad50 binding region separated from the capping domain by a flexible linker [13–17]. Rad50 belongs to the structural maintenance of chromosome (SMC) family, which are members of the ATP-binding cassette (ABC) ATPase superfamily. Rad50 monomers interact through two main interfaces. The first interface is called the  ‘Zn^2+^ hook’ and is coordinated by two absolutely conserved Cys-X-X-Cys motifs. The second, and more extensive interface, is the globular nucleotide binding domain (NBD). This domain is composed of the Walker A, Walker-B, and signature motifs; the Q and D loops; and the ‘basic switch’ (H-loop) [18,19]. The portion of the Rad50 dimer (and SMC proteins) that corresponds to the transmembrane domain of transporter ABC ATPases exists in the form of coiled coils. This domain can reach lengths of up to 500 Å in eukaryotes but is much shorter in bacteriophage T4 [16,20,21].

The binding of adenosine triphosphate (ATP) brings together parts of the NBD in *trans,* leading to a functional ATPase site. In this ‘closed’ state, the dimerized NBDs prevent the access of DNA to the Mre11 nuclease site. There is a switch to an ‘open state’ upon ATP hydrolysis, allowing for DNA processing [20,22,23]. The closed and open states of the MR complex correspond to ‘ring-shaped’ and ‘rod-shaped’ conformations, respectively [24]. Additional contact points from the coiled coils contribute to the stabilization of the ring-shaped conformation [24–29]. SMC family members hydrolyze ATP very slowly as compared with proteins with relatively similar functions [30,31]. However, the binding of Mre11 and DNA has been shown to increase Rad50's activity by 20-fold and enhance the cooperativity between ATPase sites [24,31–33]. Rad50 also promotes the nuclease activity of Mre11, and ATP hydrolysis supports the repetitive removal of nucleotides from DNA substrates [32,33] and may facilitate the melting of double-stranded DNA (dsDNA) and entry into the nuclease site [34]. Within the context of ATP-driven DNA melting, the enhancement of ATP turnover rate would suggest a repetitive process (e.g., unwinding) or cycles of dsDNA melting and reannealing [11,34].

Mre11 and Rad50 modulate the activity of each other, suggesting the presence of allosteric communication between binding interfaces and catalytic sites. Several studies have illuminated portions of these pathways. The binding of ATP causes a structural rearrangement that culminates in the assembly of a DNA binding motif contributed by each Rad50 monomer [34]. Mutation of the signature motif has also been shown to decrease DNA activation of ATP hydrolysis, reinforcing the link between the DNA and ATP binding sites [35]. The interaction of bound ATP with the signature motif is relayed to the nuclease domain via the coiled-coils [36]. Previous work from our group has identified a set of coevolving residues from the active sites of T4 phage MR complex that are functionally linked [37]. Solution-based structural methods have revealed a map of allosterically perturbed residues extending from the basic switch, signature motif, and D-loop regions affecting ATPase as well as nuclease activities. It has been suggested that the capping domain is a possible bridge for the transmission of the structural changes between the coiled-coil and the nuclease active site [33,38].

In the present study, we set out to investigate the functioning of the MR complex in the context of DNA binding site mutations. Bioinformatics methods applied to T4 Rad50 suggest a positively charged stretch of amino acids (Arg^154^, Arg^155^, and Lys^156^) as possible DNA-interacting residues [39]. Arg^154^ is particularly interesting in that it is also one of the residues previously identified by statistical coupling methods [37]. The mutation of these residues only moderately affected the protein's affinity for DNA; however, we unexpectedly found that they promoted the enhanced ATPase state even in the absence of DNA. The mutation of a residue that may electrostatically interact with Arg^154^ and Asp^479^, partially uncoupled nuclease activity from ATP dependence. Overall, the results suggest that the activation of ATP hydrolysis by DNA resembles classic allosteric activation, rather than ATP serving as a substrate for DNA end-melting or unwinding.

## Results

### Moderate effects on DNA binding affinity

**Figure 1:**
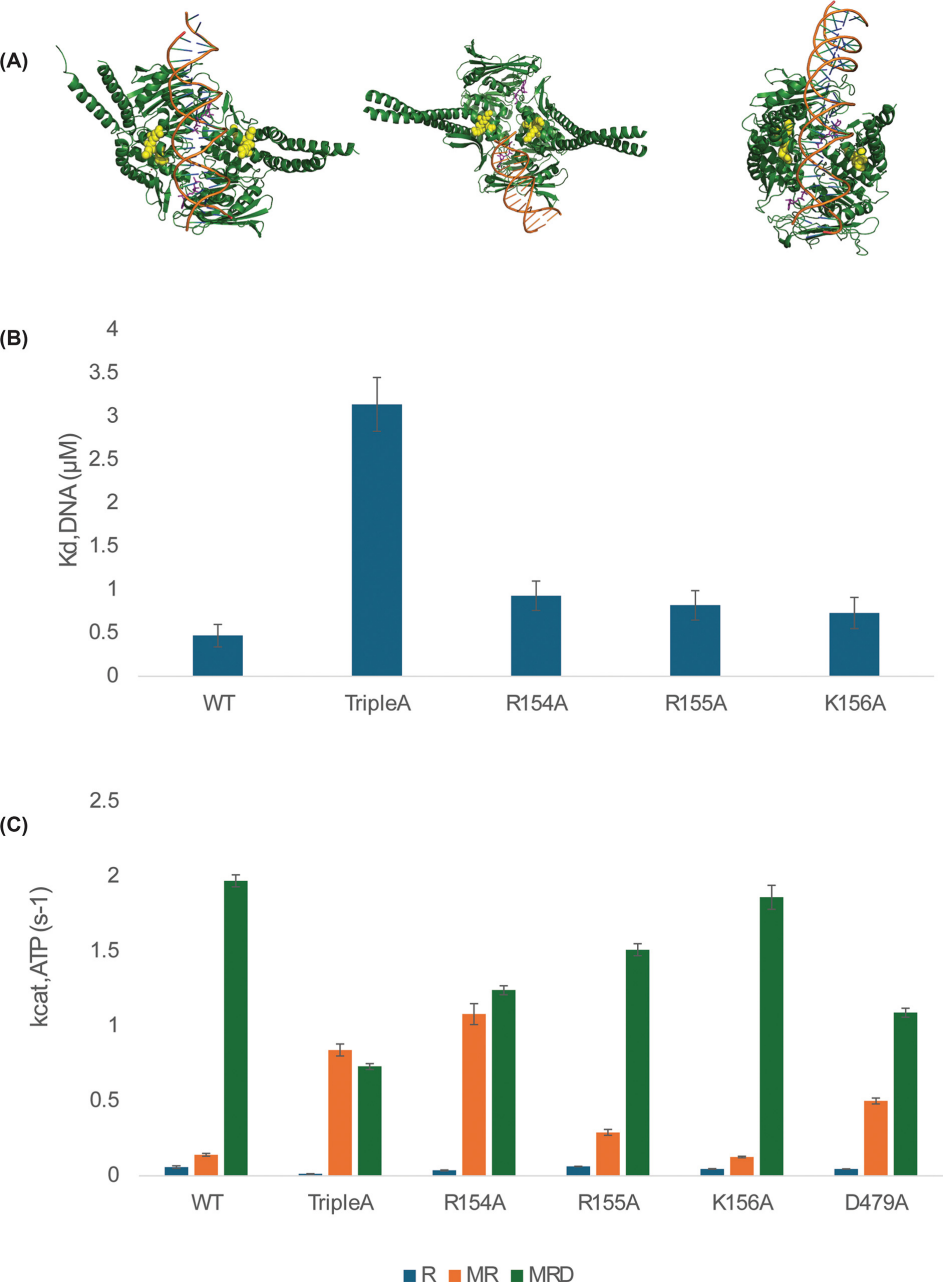
Characterization of basic-patch mutants: DNA binding and ATPase activation (**A**) Basic patch residue equivalents (yellow spheres) from crystal structures with DNA. (a) *Methanocaldococcus jannaschii* (5DNY), (b) *E. coli* (6S85), and (c) *C. thermophilum* (5DAC). (**B**) Fluorescence polarization assays for Rad50 with 5′ HEX-labeled dsDNA 50mer. TripleA showed the largest change in binding affinity with a Kd of 3.14 ± 0.31 μM as compared with 0.47 ± 0.13 μM for the WT enzyme. The *K*_d_ values for R154A, R155A, and K156A are 0.93 ± 0.17, 0.82 ± 0.17, and 0.73 ± 0.18 μM, respectively. (**C**) Constitutive ATPase activation of Rad50 mutants. TripleA and R154A display the highest activation of ATP hydrolysis activity by Mre11 alone. The largest DNA responses were shown by WT, K156A, and R155A, respectively.

Clusters of basic amino acids located in the nucleotide binding domain and the coiled-coil regions of Rad50 contribute to DNA binding in an ATP-dependent manner [34]. The Rad50 mutations introduced in the present study were chosen based on the results of sequence-based DNA binding site predictions described in Streff *et al.*, which identified the Rad50 C-terminal region as a participant in DNA binding [39]. Additionally, Arg^154^ was among the residues that were predicted by four or more of the prediction algorithms [39]. Arg^154^ had also been previously identified by statistical coupling methods as a potential allosteric residue [37]. We eliminated the positive charge of Arg154 (R154A) and its adjacent basic residues (R155A and K156A), collectively referred to as the ‘basic patch’ (Figure 1A). We also created a variant combining all three mutations (TripleA). All Rad50 variants were purified to near homogeneity and displayed circular dichroism spectra similar to that of WT-Rad50, indicating that the mutations did not grossly disrupt the overall fold (Supplementary Figures S1 and S2). For each mutant, we examined the changes in DNA binding affinity. The combined mutation of all three residues caused a moderate six-fold decrease in DNA binding affinity (a *K*_d_ of 3.1 ± 0.3 μM for TripleA versus 0.5 ± 0.1 μM for WT-Rad50). Single amino acid mutations, on the other hand, did not bring about a significant change in affinity (Figure 1B). This suggests that the residues are not likely to be involved in direct binding interactions with DNA. Although residues equivalent to the basic patch of Rad50 from other systems are positioned away from DNA binding sites, they may still contribute to DNA binding indirectly. One possibility is an allosteric route that facilitates DNA binding upon the ATP-mediated dimerization of the nucleotide binding domains of Rad50 [34].

### Mre11 activation of ATP hydrolysis

**Figure 2:**
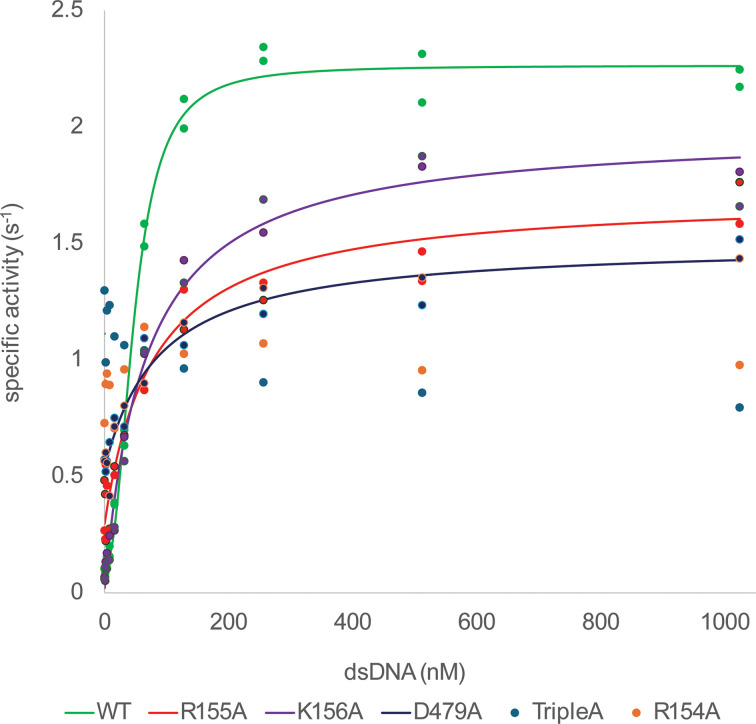
Basic-patch mutations reduce DNA-dependent cooperativity of Rad50 ATPase activation DNA activation assays for MR complexes. A plot of MR complex ATP hydrolysis activity versus dsDNA concentration. WT MR complex shows a cooperative DNA activation with *K_act_* 48 ± 2 nM. The Mre11-bound Rad50 mutants, R155A and K156A, respond non-cooperatively to DNA with *K*_act_ 83 ± 21 nM and 58 ± 4 nM, respectively. The Mre11-bound D479A Rad50 mutant shows a 24-fold increase in ATPase activity compared with Rad50 alone; however, it still displays a hyperbolic response to increased DNA concentration, with a *K*_act_ of 85 ± 25 nM. The ATPase activity of the Mre11-bound Rad50 mutants, TripleA and R154A, is insensitive to DNA across all concentrations examined.

The relatively slow ATPase activity of Rad50 is stimulated in the presence of Mre11 and dsDNA. The mutant Rad50 proteins retained basal ATPase activity, although basal kinetic parameters differed from wild type (WT) in some cases, and the addition of Mre11 and/or DNA produced varying degrees of ATPase activation (Table 1). Surprisingly, TripleA and R154A showed 70- and 30-fold ATPase activations, respectively, upon addition of Mre11 alone. This approaches the activation observed for the WT MR complex in the presence of DNA (Figure 1C and Table 1). A similar pattern is present when ATPase stimulation is examined under increasing concentrations of DNA (Figure 2 and Table 2). WT-Rad50 has a sigmoidal pattern of dsDNA stimulation of ATPase activity with a K_activation_ (*K*_act_) value of 48 ± 2 nM and a Hill coefficient of 2.3 ± 0.2 (the Hill coefficient is a measure of cooperativity; values greater than 1 indicate positive cooperativity between binding sites) (Table 2). R155A and K156A displayed reduced DNA activation cooperativity compared with the WT enzyme (Hill coefficients of 1.1 and 1.5, respectively). TripleA and R154A are relatively insensitive to increases in DNA concentration, and their ATPase activities approach that of the fully activated WT enzyme, suggesting that these mutations shift the complex toward an ATPase-active-like state.

**Table 1:** Kinetic parameters for the ATPase activities of WT-Rad50 and basic patch or D479 mutations

Protein^a^	*k*_cat_,_ATP_ (s^−1^)	*K*_M_,_ATP_ (μM)	Hill coeff.
**R_WT_**	0.06 ± 0.01	31 ± 6	1.3 ± 0.2
**R_TripleA_**	0.012 ± 0.002	1600 ± 600	1.1 ± 0.2
**R_R154A_**	0.036 ± 0.003	700 ± 100	1.2 ± 0.1
**R_R155A_**	0.062 ± 0.002	23 ± 2	1.6 ± 0.1
**R_K156A_**	0.045 ± 0.002	19 ± 1	1.5 ± 0.1
**R_D479A_**	0.045 ± 0.001	48 ± 2	2.2 ± 0.2
**MR_WT_**	0.14 ± 0.01	30 ± 5	1.3 ± 0.2
**MR_TripleA_**	0.84 ± 0.04	260 ± 20	1.5 ± 0.1
**MR_R154A_**	1.1 ± 0.1	130 ± 15	1.7 ± 0.1
**MR_R155A_**	0.29 ± 0.02	100 ± 15	1.4 ± 0.2
**MR_K156A_**	0.13 ± 0.01	36 ± 3	1.4 ± 0.1
**MR_D479A_**	0.50 ± 0.02	210 ± 14	1.7 ± 0.1
**MRD_WT_**	1.97 ± 0.04	67 ± 2	2.3 ± 0.1
**MRD_TripleA_**	0.73 ± 0.02	299 ± 13	2 ± 0.2
**MRD_R154A_**	1.24 ± 0.03	90 ± 3	2.2 ± 0.1
**MRD_R155A_**	1.51 ± 0.04	90 ± 4	2.1 ± 0.1
**MRD_K156A_**	1.9 ± 0.1	89 ± 6	1.8 ± 0.1
**MRD_D479A_**	1.09 ± 0.03	200 ± 9	2.3 ± 0.2

a Rad50 (R), Rad50+Mre11 (MR), and Rad50+Mre11+dsDNA (MRD) conditions.

**Table 2:** DNA activation of WT-Rad50 and basic patch or D479 mutations

Protein	DNA activation^a^	*K_act_*_,DNA_ (nM)	Hill coeff.
**R_WT_**	18	48 ± 2	2.3 ± 0.2
**R_TripleA_**	0.6	NA	NA
**R_R154A_**	1.3	NA	NA
**R_R155A_**	5	83 ± 21	1.1 ± 0.3
**R_K156A_**	19	58 ± 4	1.5 ± 0.2
**R_D479A_**	3	85 ± 25	1.0 ± 0.3

a The ratio of the maximal activity at saturating DNA/activity in the absence of DNA.

Given the significantly increased *K*_M_,_ATP_ values for the basal activity of R154A and TripleA (Table 1), it is likely that this basic patch is somehow involved in ATP binding and/or hydrolysis. Addition of Mre11 lowers the mutant’s K_M,ATP_ to the wild-type range, while dsDNA returns the Hill coefficient to wild-type cooperativity, suggesting that these Rad50 mutants bind both Mre11 and DNA. Based on a homology model of T4 Rad50 built using the crystal structures of *Methanocaldococcus jannaschii* and *Chaetomium thermophilum* Rad50 as templates (PDB accession codes 3AV0 and 5DAC), Asp479 is positioned to possibly participate in ionic interactions with the basic-patch residues [36,40]. The D479A mutant protein has a high cooperativity for ATP hydrolysis regardless of the addition of Mre11 and DNA (Table 1). On the other hand, it does not display the cooperative DNA activation shown by WT; it instead follows a hyperbolic pattern that is similar to R155A and K156A (Table 2). Even though R155A and D479A are somewhat sensitive to increasing DNA concentrations (DNA activation of five- and three-fold, respectively), their ATPase activity is significantly activated even in the absence of DNA (Figures 1C and 2). The catalytic mechanism of T4-Rad50 has previously been investigated, and structural pathways have been proposed for the enhanced cooperativity in the Mre11/Rad50/dsDNA (MRD) complex [41]. The Asp^479^ mutation is likely to perturb one of these allosteric routes, shifting the conformational equilibrium toward an ATPase-active-like state.

### ATP hydrolysis uncoupled from enhancement of nuclease activity

**Figure 3:**
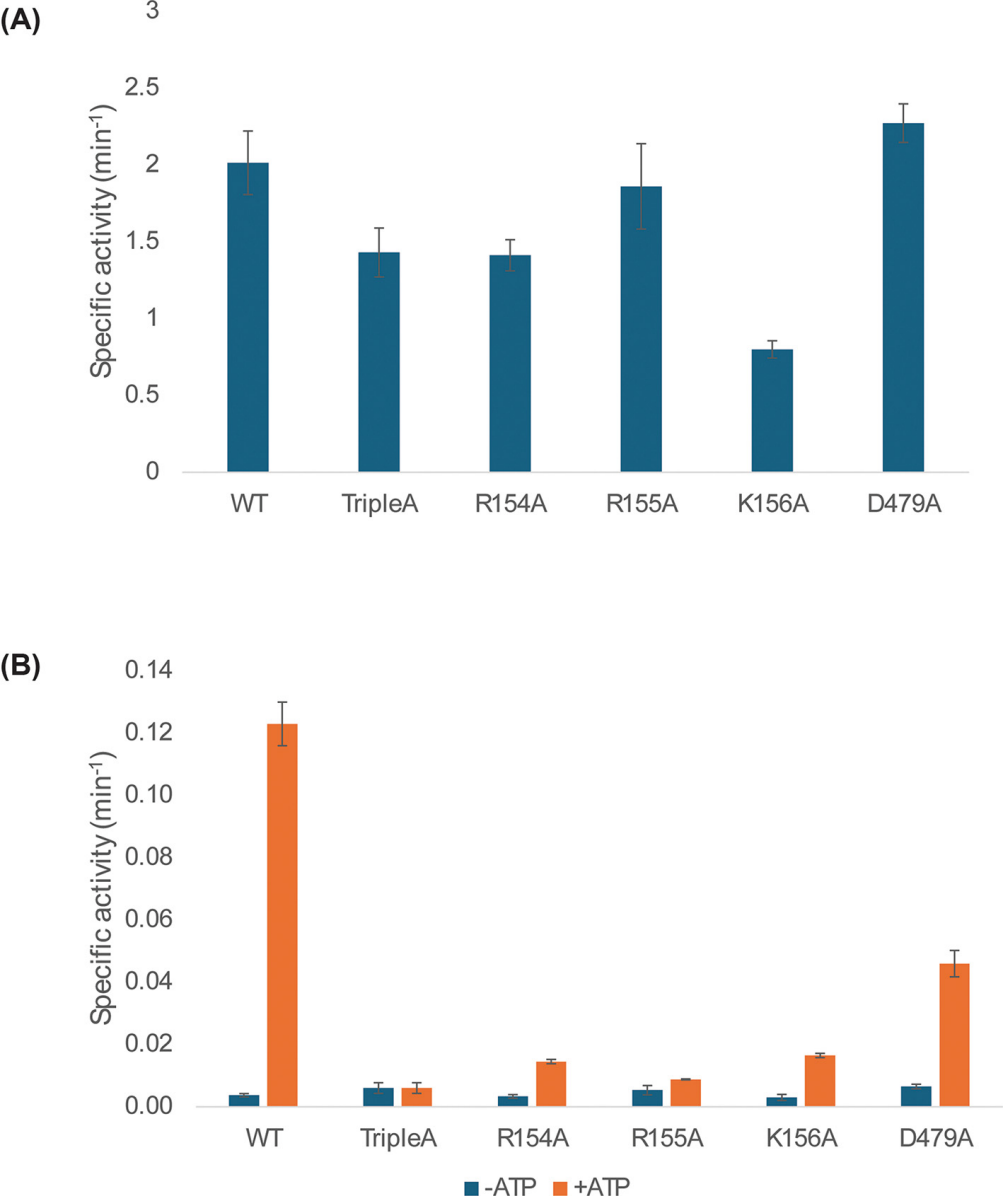
Exonuclease activity of WT-Mre11 with WT- and mutant Rad50 proteins (**A**) Exonuclease activity on the 1-position 2AP DNA in the absence of ATP. (**B**) Exonuclease activity on the 18-position 2AP DNA in the presence and absence of ATP.

**Figure 4:**
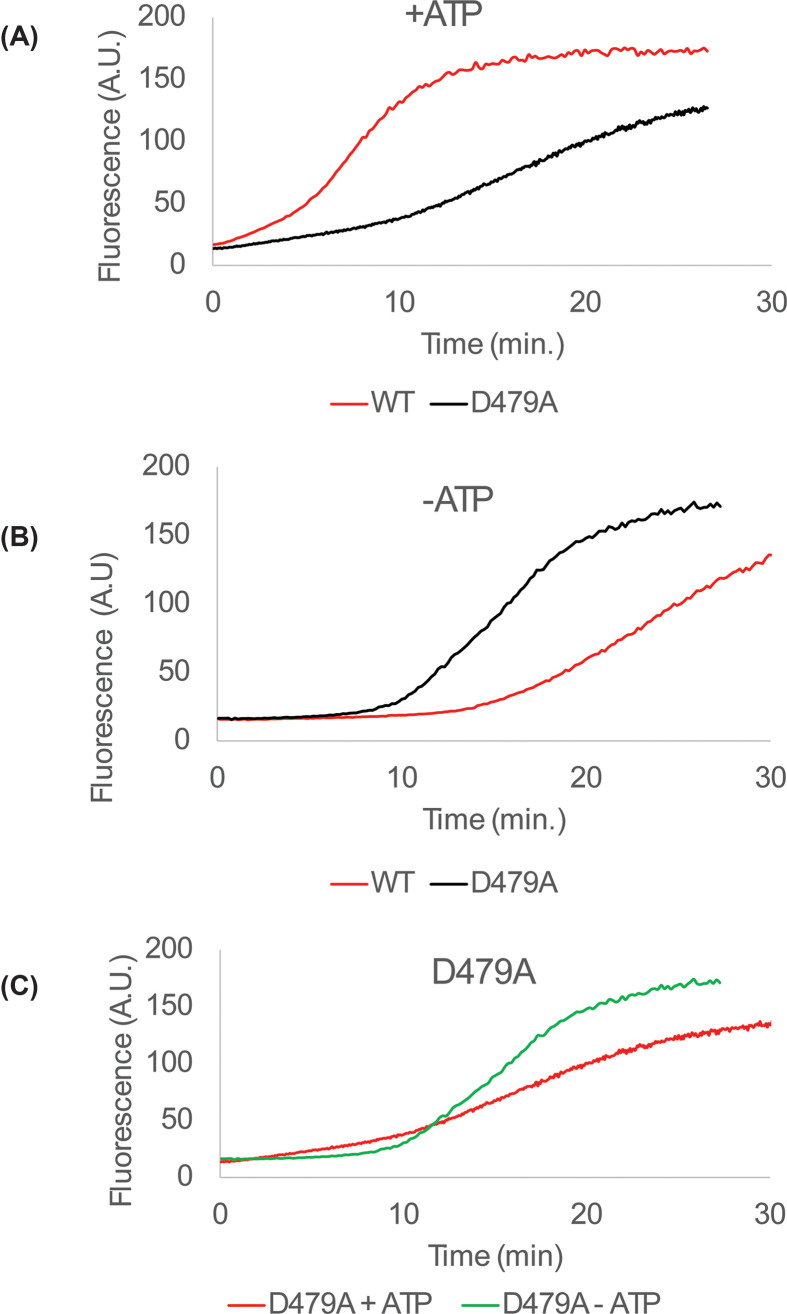
Exonuclease time courses with the 18-position 2AP DNA using WT-Mre11 with WT or the D479A mutant Rad50 proteins The exonuclease reactions of WT and D479A in the absence (**A**) or presence (**B**) of ATP. (**C**) The exonuclease reactions of D479A in the absence and presence of ATP.

The formation of the MR complex through the binding of Rad50 to Mre11 significantly enhances the rate of 3′ nucleotide excision from dsDNA, even in the absence of ATP, compared with the activity exhibited by Mre11 alone. However, ATP binding and hydrolysis are required to enable the processive exonuclease activity of Mre11 along the DNA substrate [15,32,42]. Rad50 stimulation of the initial DNA cleavage event appeared to be normal across all the mutant proteins investigated (Figure 3A). However, ATP hydrolysis did not support processive exonuclease activity at WT levels. This uncoupling of ATP hydrolysis and exonuclease activity was most pronounced with the TripleA mutant, which displays virtually no activation at the early stages of the reaction as compared with the 31-fold activation of WT Rad50 (Table 3 and Figure 3B). MRD-D479A also has normal exonuclease activity using the 1-position 2AP DNA (Figure 3A) and displays some degree of the ATP-dependent decrease of the lag phase that is observed in the 18-position DNA assays (Figures 3B and 4). However, in sharp contrast to WT and the other Rad50 mutants, the full-time course of 18-position nuclease assays reveals that D479A supports higher nuclease activities in the absence of ATP (Figure 4).

**Table 3:** Initial exonuclease activity rates from 1- and 18-position nuclease assays

	Specific nuclease activity (min^−1^)			
	1-pos	18-pos (-ATP)	18-pos (+ATP)	ATP activation
**WT**	2.0 ± 0.2	0.004 ± 0.0005	0.123 ± 0.007	31
**TripleA**	1.4 ± 0.2	0.006 ± 0.0017	0.006 ± 0.002	1
**R154A**	1.4 ± 0.1	0.003 ± 0.001	0.015 ± 0.001	5
**R155A**	1.9 ± 0.3	0.005 ± 0.002	0.009 ± 0.0002	2
**K156A**	0.8 ± 0.06	0.003 ± 0.001	0.017 ± 0.001	6
**D479A**	2.3 ± 0.1	0.006 ± 0.001	0.046 ± 0.004	8

## Discussion

The slow ATPase activity of Rad50 is enhanced by Mre11 and dsDNA [32], but the structural details of how this activation occurs remain incompletely understood [43]. In the present study, we show that a basic patch near the base of the T4-Rad50 coiled-coils contributes to this process in a manner that is distinct from simple direct DNA binding. Although Arg154, Arg155, and Lys156 were initially selected based on DNA-binding site predictions, the mutation of these residues caused only modest changes in DNA affinity, with the largest effect observed for the TripleA mutant and comparatively little effect for the individual substitutions. This suggests that the basic patch is not likely to function primarily as a direct DNA-contact surface. Instead, the biochemical data indicate that this region participates in communication between ligand binding and the ATPase machinery of Rad50. In particular, R154A and TripleA displayed strong ATPase activation in the presence of Mre11 alone, approaching the level normally observed for the WT-MR complex only after the addition of DNA. Overall, the results support the idea that the basic patch contributes to an allosteric route linking Mre11 and DNA engagement with productive ATP hydrolysis.

The uncoupling of ATPase activity from the enhancement of repetitive nucleotide excision implies that this allosteric route has been perturbed by the mutations. All of the Rad50 variants supported near-WT stimulation of the initial cleavage event in the 1-position assay, suggesting that MR complex formation and promotion of the first nuclease step remain largely intact. On the other hand, ATP-dependent stimulation of long-range cleavage in the 18-position assay was reduced for all of the mutants, with the most pronounced defect observed for TripleA. Critically, this uncoupling demonstrates that elevated ATP turnover alone is not sufficient to support robust processive nuclease activity: if ATP were simply serving as a substrate for DNA end-melting or unwinding, then the enhanced ATPase activity of R154A and TripleA should have supported, rather than failed to support, processive cleavage. It is likely that ATP turnover must be coupled to the conformational changes that allow DNA to be productively engaged during repeated cleavage events. In this regard, the results suggest that DNA activation of the T4 MR ATPase resembles a form of allosteric activation, rather than ATP simply serving as a substrate for DNA melting or unwinding.

At present, the data do not allow the assignment of these mutants to a single discrete structural state. Our experiments do not directly define the conformations of the ATPase heads, the coiled-coils, or the nuclease gate. The most conservative interpretation is that mutation of the basic patch alters the conformational equilibrium of the complex, favoring an ATPase-active-like state that can be reached through Mre11 binding even in the absence of DNA, but that does not efficiently support the ATP-dependent transitions required for processive exonuclease activity. One possibility is that the affected step involves DNA rearrangements needed for repeated cleavage, although the present data do not distinguish whether this reflects local dsDNA melting, positioning of the 3′ strand in the nuclease site, or another ATP-dependent conformational change. This interpretation is consistent with proposals for post-hydrolysis MR intermediates that support long-range resection [44] and with recent structural and dynamics data showing that DNA-stimulated Rad50 ATP hydrolysis is linked to specific conformational states of the MR-DNA complex [43], while remaining compatible with evidence that Mre11 can accommodate relatively undistorted dsDNA [24,34]. Several features of the data support this view. First, R154A and TripleA show strong ATPase activation with Mre11 alone. Second, both mutants are relatively insensitive to increasing DNA concentrations. Third, the elevated basal K_M,ATP_ values of these mutants become more WT-like in the presence of both Mre11 and DNA, suggesting altered conformational energetics rather than a loss of the relevant binding interfaces. Framed in this way, the results support a change in the conformational landscape of the MR complex but do not require the stronger conclusion that these mutations stabilize a true DNA-bound state.

**Figure 5:**
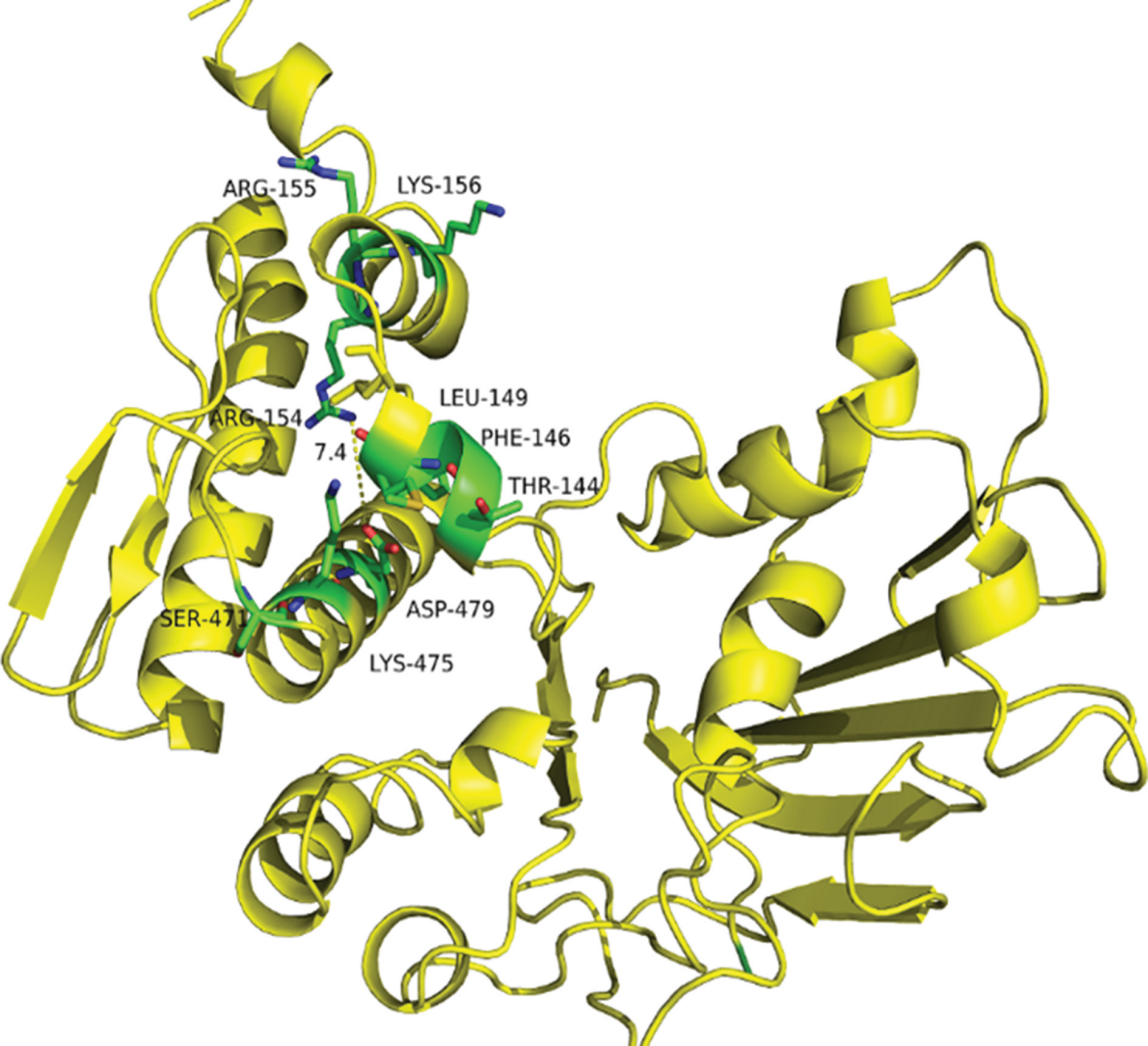
A comparison of the signature motif and signature coupling residues of P. furiosus Rad50 with proposed counterparts on the T4-Rad50 modeled structure Structural homology modeling was performed by Phyre^2^ with 100% coverage at >90% confidence.

The D479A mutant further supports the presence of long-range allosteric coupling but also indicates that not all perturbations within this region act in an identical manner [37]. D479A partially phenocopies the basic-patch mutants in that it shows elevated ATPase activity in the absence of DNA and loss of the cooperative DNA activation observed for WT. However, the effect on nuclease activity is distinct, particularly in the modestly elevated nuclease activity observed in the absence of ATP relative to WT and the residual ATP responsiveness retained in the 18-position assay. It is therefore likely that Asp479 affects the same general regulatory pathway as the basic patch, but through a different mechanistic contribution. Based on the homology model, Asp479 may participate in nucleotide-dependent electrostatic interactions with Arg154 and/or Arg155 near the base of the coiled coils (Figure 5). However, this should presently be considered a working hypothesis rather than a demonstrated structural mechanism.

It has previously been shown that nucleotide state is communicated through Rad50 to Mre11 and that perturbations within the ATPase domain can alter both ATP hydrolysis and nuclease activity [36,40,44,45]. The present results are also consistent with the possibility that the basic-patch mutations perturb the network of basic-switch and salt-bridge interactions that link nucleotide binding to movements of the coiled-coils and the Mre11 interface [36,44]. Within this framework, the present results support a model in which electrostatic interactions extending from the Rad50 nucleotide-binding domain toward the base of the coiled-coils and the Mre11 interface help coordinate ATPase activation with nuclease output [25]. In the T4 system, the basic patch and Asp479 may represent a species-specific variation on this general allosteric theme. It is likely that this region helps tune the conformational transitions by which Mre11 and DNA promote a catalytically productive ATPase cycle and couple ATP turnover to processive DNA cleavage. Structures of the T4-MR complex in nucleotide-free and nucleotide-bound states, along with additional dynamics-based experiments, will be required to determine whether this pathway involves direct Asp479-basic patch interactions, altered coiled-coil positioning, changes at the Mre11 linker interface, or a combination of these features.

## Conclusions and perspectives

The present study identifies a basic patch on bacteriophage T4 Rad50, comprising Arg154, Arg155, and Lys156, as a contributor to the allosteric pathway that couples Mre11 and DNA engagement to productive ATP hydrolysis and processive nuclease activity. The results demonstrate that enhanced ATPase activity alone is not sufficient to drive processive DNA cleavage, pointing to a requirement for specific ATP-dependent conformational transitions that the basic patch helps to gate. The nearby residue Asp479 participates in the same general pathway but through a distinct mechanistic contribution, consistent with a network of interacting residues rather than a single linear allosteric route. Together, these findings strengthen the view that DNA acts as an allosteric effector of the MR complex and identify a previously unrecognized pathway linking the Rad50 nucleotide-binding domain with nuclease regulation [31,34]. Future structural studies of the T4 MR complex in defined nucleotide states, combined with solution-based dynamics experiments, will be needed to establish the precise geometry of these interactions and to determine how broadly this allosteric mechanism is conserved across the MR family.

## Methods

### Mutation, expression and purification of proteins

Rad50 mutations were introduced using the Phusion Site-Directed Mutagenesis Kit (Thermo Fisher) and confirmed through plasmid sequencing. Wild-type (WT) and mutant Rad50 and Mre11 proteins were expressed in *E. coli* BL21(DE3) as C-terminal fusions with an intein/chitin-binding domain (pTYB1 vector, New England Biolabs) and purified as described previously [32,35]. Briefly, cells were lysed by sonication in 20 mM Tris–HCl, 500 mM NaCl, 1 mM EDTA, pH 8.0, and the clarified lysate was loaded onto a chitin-affinity column. Following an overnight wash, intein self-cleavage was induced by incubation in cleavage buffer containing 75 mM β-mercaptoethanol for 16 h at 4°C. Tag-free Mre11 was dialyzed into storage buffer (20 mM Tris–HCl, 200 mM NaCl, 20% glycerol, pH 8.0) and stored at −80°C. Tag-free Rad50 was further purified by phosphocellulose (P11) chromatography, eluting with 400 mM NaCl, and stored at −80°C. Purity of WT Rad50, along with the mutant proteins and Mre11, was confirmed using 10% sodium dodecyl sulfate–polyacrylamide gel electrophoresis (Supplementary Figure S1). Each protein was subjected to circular dichroism (CD) spectroscopy to confirm that all mutant proteins retain the overall fold of WT (Supplementary Figure S2). CD was carried out at room temperature (∼22°C) on a MOS-500 spectropolarimeter from Biologic in a 0.1 cm cell using a protein concentration of 0.05 mg/ml. Spectra were collected from 200 to 260 nm in increments of 1 nm. Each spectrum was blank-corrected and normalized to the ellipticity of the WT protein at 216 nm to correct for minor differences in protein concentration.

### Fluorescence polarization assay

DNA binding affinities for WT and mutant Rad50 proteins were determined using a 5′-hexachlorofluorescein labeled dsDNA substrate. 100 nM of DNA was titrated with 0-3 μM of Rad50 in buffer containing 50 mM Tris–HCl, 50 mM KCl, 10 mM MgCl_2_, and 0.1 mg/ml bovin serum albumin, pH 7.6. This buffer composition was maintained for all subsequent experiments in the present study. Fluorescence polarization values were measured using a Synergy 2 Multi-Mode Microplate Reader. The data was fitted to the simple binding mechanism R + D* <==> R.D*, where R = Rad50 and D* is labeled DNA (DynaFit version 4.07.128, BioKin Ltd). All fluorescence polarization experiments were performed in triplicate (*n* = 3 independent experiments), and reported errors represent standard deviations.

### ATP hydrolysis assays

The ATP hydrolysis activities of Rad50 alone, as well as in complex with Mre11 and dsDNA, were measured using enzyme-coupled ATPase assays. Various concentrations of ATP were pre-incubated at room temperature for 5-10 min in reaction buffer containing 1 mM phosphoenolpyruvate, 0.1–0.2 mM reduced nicotinamide adenine dinucleotide, 6.7–8.1 U/ml pyruvate kinase, and 10–12.1 U/ml lactate dehydrogenase. The pre-incubation helps convert any adenosine diphosphate that may have been present into ATP. dsDNA, whenever present, was held in excess and at least 10-fold above the *K_act_* (1 μM). Reactions were started with Rad50 (1, 2, and 4 μM), MR (0.2, 0.5, and 1 μM), and MRD (0.05 and 0.15 μM) and monitored at 30°C in a Varian Cary Eclipse Fluorescence Spectrophotometer. Mre11 was held at a 1.25-fold excess over Rad50 to ensure that all of the Rad50 was in the MR complex. Specific activities were calculated from linear portion of the progress curves at individual ATP concentrations. The combined data was fitted to the Hill equation, and kinetic parameters were extracted (Sigmaplot 14, Systat Software Inc.). Each ATPase condition was measured in two independent experiments, with multiple ATP concentrations per experiment. Kinetic parameters and their associated errors represent the best-fit values and standard errors from nonlinear regression of the combined datasets.

To investigate the level of activation exerted by DNA on the ATPase activity of the Rad50 proteins, fixed ATP (1 mM) assays were done with 0.1 μM MR and dsDNA concentrations ranging from 0 to 32 nM. Whenever possible, the data were fitted to a modified Hill equation: *f* = (*V*m*X^*n*^)/(*K*_act_^*n*^+ *X^n^*) + *C*, where *K*_act_ is the constant for DNA activation and *C* represents baseline activation. DNA activation assays were performed in two independent experiments. Reported *K*_act_ and Hill coefficient values and their errors represent best-fit values and standard errors from nonlinear regression of the combined datasets.

### Nuclease activity assays

Initial and repetitive nucleotide excisions were measured using 2-aminopurine (2AP)-labeled oligonucleotide substrates [15,32]. The 2AP label was placed at the 1- or 18-position relative to the 3′ end of the top strand. The 3′ end of the bottom strand had a phosphorothioate linkage to prevent nuclease activity on that strand. The MR complex was held at 0.05 and 0.4 μM for 1- or 18-position assays, respectively. DNA was 1.3 μM, and Rad50 was held in a 1.1-fold excess over Mre11. The reaction contained 0.3 mM MnCl_2_ and 18-position assays were performed in the presence or absence of 1 mM ATP. The reactions were allowed to proceed for 10–60 min at 30°C, and specific activities were calculated from the slope of the first 5 min of the progress curves. All nuclease assays were performed in triplicate (*n* = 3 independent experiments), and reported errors represent standard deviations.

#### Oligonucleotide sequences

Fluorescence polarization and nuclease assays:

50mer_F (1-position 2AP) 5′-ATGGTGTGCTCGTCCGCGATTAGTAGGCATAGAGACAGATACAGCGACA(2AP)-3′
50mer_F (18-position 2AP) 5′-ATGGTGTGCTCGTCCGCGATTAGTAGGCAT(2AP)GAGACAGATACAGCGACAA-3′
50mer_R 5′-TTGTCGCTGTATCTGTCTCTATGCCTACTAATCGCGGACGAGCACACCAT-3′

ATPase assays:

50mer_F 5′-CTCTTGGTGATTATGATGGTTGCAATACATTTAATTTCATTATCAATTAG-3′
50mer_R 5′-CTAATTGATAATGAAATTAAATGTATTGCAACCATCATAATCACCAAGAG-3′

## Supplementary Material

Supplementary Figures S1-S2

## Data Availability

All data published as part of this submission will be made available upon reasonable request.

## Open Access

Open access for this article was enabled by the participation of Iowa State University in an all-inclusive Read & Publish agreement with Portland Press and the Biochemical Society.

## Abbreviations

2AP: 2-aminopurine
ABC: ATP-binding cassette
ATP: adenosine triphosphate
CD: circular dichroism
DSB: DNA double-strand break
dsDNA: double-stranded DNA
HR: homologous recombination
MR: Mre11/Rad50 complex
MRD: Mre11/Rad50/dsDNA complex
NBD: nucleotide-binding domain
PDB: Protein Data Bank
SMC: structural maintenance of chromosomes
ssDNA: single-stranded DNA
WT: wild type
