# Supplementary material for: Basic-patch mutations in bacteriophage T4 Rad50 uncouple ATPase activation from processive nuclease activity
Source: Biosci Rep. 2026 Jul 23;46(8):BSR20260367. doi: 10.1042/BSR20260367 (PMC13402864; doi:10.1042/BSR20260367)
Supplement: Supplementary Figures S1-S2 [file BSR-2026-0367_supp.pdf]

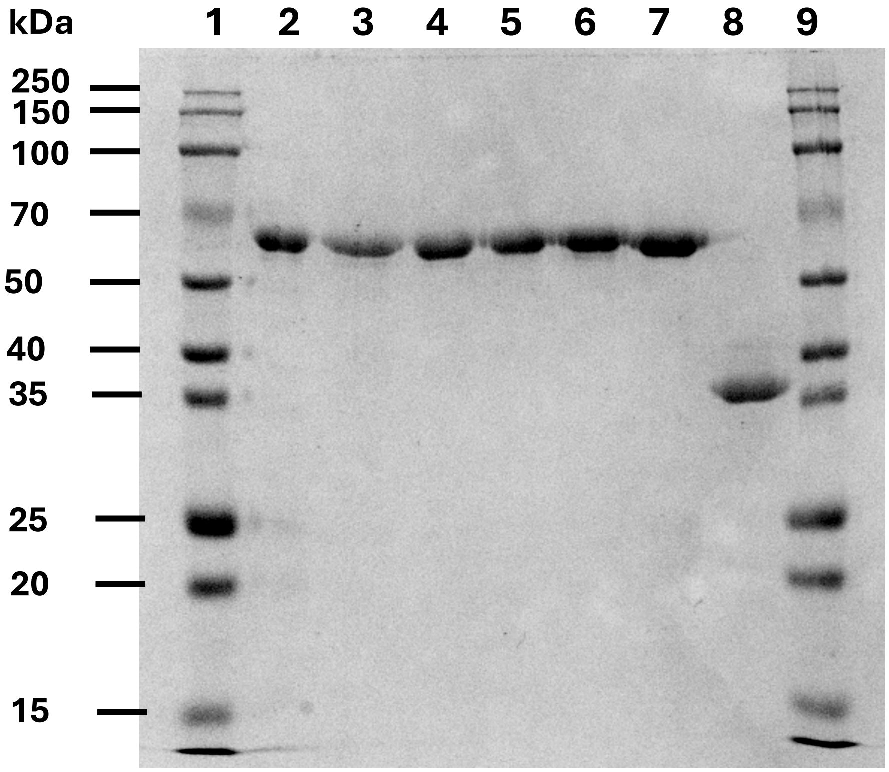

Supplemental Figure S1. SDS-PAGE analysis of Rad50 proteins and WT-Mre11. The lanes contain: (1) molecular weight marker with mass indicated along with left side of the gel, (2) WT-Rad50, (3) R154A-Rad50, (4)R155A-Rad50, (5) K156A-Rad50, (6) TripleA-Rad50, (7) D479A-Rad50, (8) WT Mre11, (9) molecular weight marker

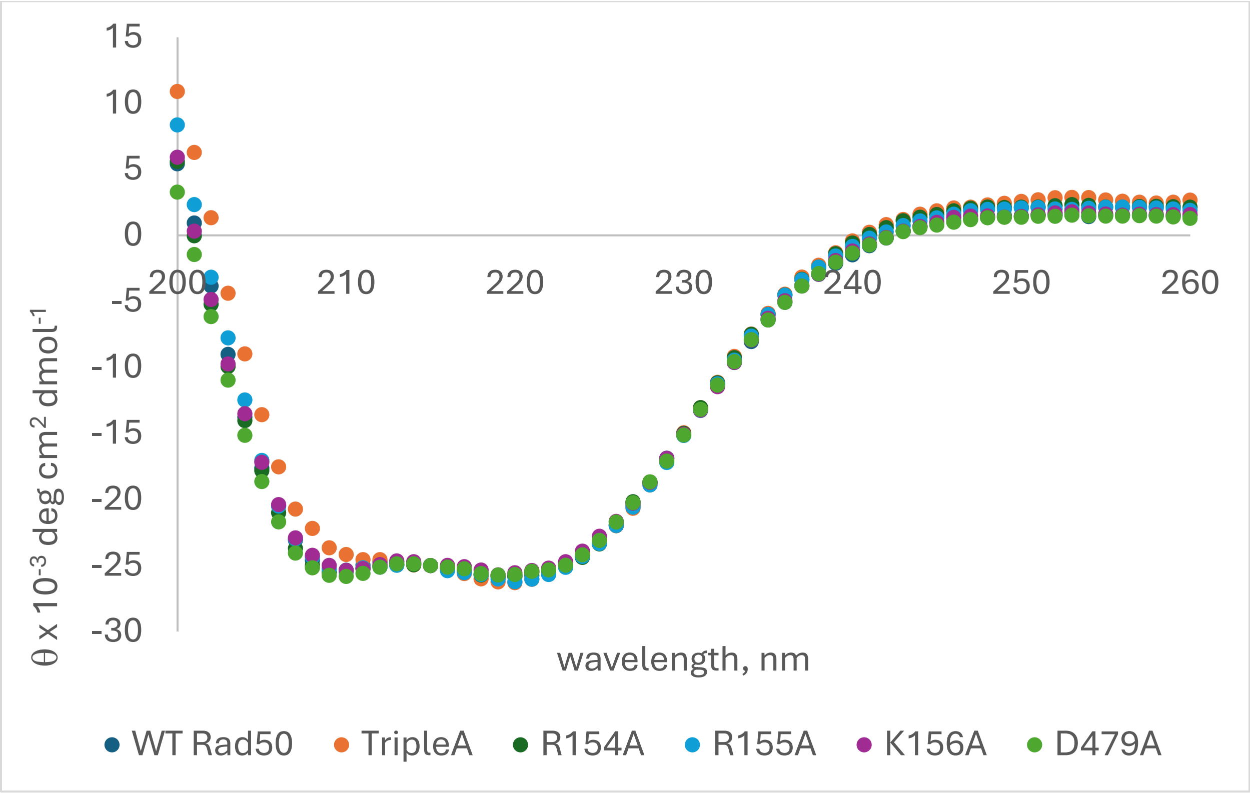

Supplemental Figure S2. Circular Dichroism spectra of WT and mutant Rad50 proteins.
